# Safety Evaluation of *Yukmijihwang-tang*: Assessment of Acute and Subchronic Toxicity in Rats

**DOI:** 10.1155/2011/672136

**Published:** 2010-12-22

**Authors:** Hyekyung Ha, Jun Kyoung Lee, Ho Young Lee, Woo Suk Koh, Chang Seob Seo, Mi-Young Lee, Dae Sun Huang, Hyeunkyoo Shin

**Affiliations:** ^1^Herbal Medicine EBM Research Center, Korea Institute of Oriental Medicine, Daejeon 305-811, Republic of Korea; ^2^Division of Toxicology, Korea Institute of Toxicology, Daejeon 305-323, Republic of Korea

## Abstract

*Yukmijihwang-tang* (YMJ; *Liu wei di huang tang* (China), *Rokumigan* (Japan)) has been used in the treatment of diseases including renal disorder, cognitive vitality, and diabetes mellitus. However, there is very little information regarding the toxicity of YMJ to give an assurance of safety for clinical treatment. To provide safety information for YMJ, we evaluated its acute and sub-chronic toxicity in rats. The single-dose toxicity of YMJ was examined using Sprague-Dawley rats. Rats were treated with YMJ extract orally at 0, 500, 1000, or 2000 mg/kg body weight. After a single administration, clinical signs were observed every day for two weeks, and body weights were measured five times, including an initial measurement on day 1 (the day of administration). In the sub-chronic oral toxicity study, YMJ was administered to rats at 0, 500, 1000, or 2000 mg/kg/day for 13 weeks. Mortalities, clinical signs, body weight changes, food and water consumption, ophthalmologic findings, urinalysis, hematological and biochemical parameters, gross findings, organ weights, and histological examination were monitored during the study period. We found no mortality and no abnormalities in clinical signs, body weights, and necropsy findings for any of the animals in the acute and sub-chronic studies following oral administration in the rat at up to 2000 mg/kg/day YMJ. YMJ may not have any single-dose toxicity; the LD_50_ of YMJ was over 2000 mg/kg, and it is safe for rats. The no-observed-adverse-effect-level (NOAEL) was considered to be 2000 mg/kg/day.

## 1. Introduction

*Yukmijihwang-tang* (YMJ; *Liu wei di huang tang *(China), *Rokumigan* (Japan)) is a herbal formula that includes six herbs: Rehmanniae Radix Preparata, Corni Fructus, Dioscoreae Rhizoma, Moutan Cortex, Poria Sclerotium, and Alismatis Rhizoma [1]. YMJ is one of the most commonly used herbal formulas for body enrichment and is widely used in traditional medicine in Korea, China, and Japan for kidney disorders such as pollakiuria (urination at short intervals) and edema [1, 2]. It was reported that YMJ was the first herbal formula to be used in traditional Korean medicine [3]. In China, YMJ had the greatest market share in traditional Chinese medicine, and the market size was about US$633 million in 2009 [4]. Recently, reports have shown that YMJ is effective for antiaging treatment, antioxidant and free radical scavenging activities [5], memory enhancement and prevention of neuronal degeneration [6, 7], and diabetes mellitus treatment [8]. 

Medicinal herbs and herbal formulas are generally considered to be safer than conventional drugs. However, there is no evidence for the quality, safety, and efficacy of most commonly used herbal formulas including YMJ. In addition, very little information exists on the safety of YMJ for clinical use.

To establish evidence-based toxicity data for YMJ, we analyzed the literature, efficacy, safety, and chemical constituents of YMJ. As a part of the safety evaluation of YMJ, acute oral dose toxicity and a subchronic oral dose toxicity studies were conducted to investigate the potential toxicity after a single or 13-week repeated oral dose administration of YMJ in Sprague-Dawley rats. The present study was carried out in compliance with good laboratory practice (GLP) and the test guidelines of the Organization for Economic Cooperation and Development (OECD) [9] and Korea Food and Drug Administration (KFDA) [10, 11].

## 2. Materials and Methods

### 2.1. Preparation of Yukmijihwang-Tang (YMJ) Extract

The constituents of YMJ are listed in Table 1. Six constituents of YMJ (20.64 kg) were chopped, mixed, and extracted with water at 100°C for 120 min in an Herb Extractor (COSMOS-660, KyungSeo Machine Co., Inchon, Korea) and filtered. The aqueous extract was lyophilized using a freeze dryer (PVTFD100R, Ilshin Lab. Ltd., Korea) and yielded 5.57 kg of brown powder (yield = 27.0%). Powders of YMJ extract were stored at 4°C.

### 2.2. Analysis of Chemical Contents from YMJ by HPLC

The chemicals used for the identification and quantification of compounds within the YMJ extract include the following: 5-hydroxymethyl-2-furaldehyde (5-HMF, MW: 126.11) as a component of Rehmanniae Radix Preparata; loganin (MW: 390.38) of Corni Fructus; paeoniflorin (MW: 480.46) and paeonol (MW: 166.17) of Moutan Cortex.

**Figure 1:**
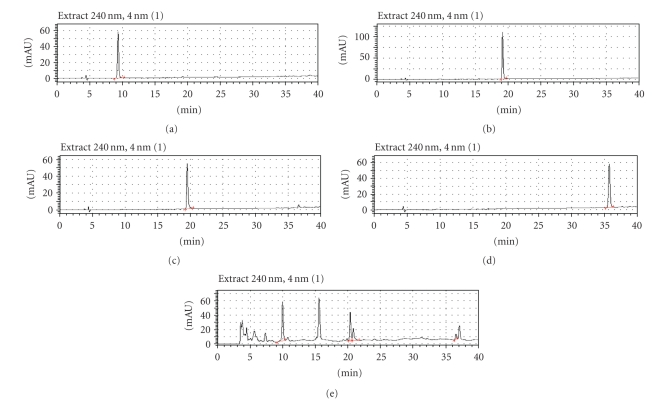
Chromatograms of standard components and *Yukmijihwang-tang*. The three major component herbs of *Yukmijihwang-tang* and their main compounds were subjected to HPLC. The chromatograms were obtained at a wavelength of 240 nm. 5-HMF ((a), retention time (RT): 8.893 min), loganin ((b), RT: 18.850 min), paeoniflorin ((c), RT: 19.188 min), paeonol ((d), RT: 35.604 min), and *Yukmijihwang-tang* extract (e).

YMJ extract (10 mg) was dissolved in 1 mL of 50% methanol and filtered through a 0.45-*μ*m syringe filter before injection into the HPLC system. The HPLC system was composed of an SCL-10A system controller, LC-6AD pump, SPD-M10A diode array detector (Shimadzu, Kyoto, Japan), and Phenomenex Luna C18 (5 *μ*m, 4.6 mm × 250 mm) column. A gradient solvent system of methanol (A) and water (B) was used as follows: 20% A/80% B (start), 50% A/50% B (10–20 min), and 100% A (40–60 min) at a flow rate of 0.7 mL/min. The chromatograms were obtained at a wavelength of 240 nm (Figure 1). 

In order to make standard calibration curves, 5-HMF (1 to 500 *μ*g/mL), loganin (0.1 to 100 *μ*g/mL), paeoniflorin (1 to 100 *μ*g/mL), and paeonol (2.5 to 50 *μ*g/mL) were diluted in 50% methanol and injected into the HPLC to give individual chromatograms. The calibration curves were plotted by calculating the peak area ratio, which has a relatively smaller error than the peak height ratio. The fitting equations of each calibration curve were as follows: 5-HMF, *y* = 14905*x* − 70874 (*r* ^2^ = 0.9998); loganin, *y* = 25362*x* − 3955.8 (*r* ^2^ = 0.9999); paeoniflorin, *y* = 15270*x* − 10013 (*r* ^2^ = 0.9998); paeonol, *y* = 19355*x* − 2039.1 (*r* ^2^ = 0.9996). The contents of the chemicals in YMJ extract were measured as the following: 5-HMF 3.70 ± 0.11 mg/g, loganin 1.77 ± 0.05 mg/g, paeoniflorin 1.08 ± 0.03 mg/g, and paeonol 1.98 ± 0.02 mg/g.

### 2.3. Animals and Housing Conditions

For the acute and subchronic toxicity studies, specific pathogen-free Sprague-Dawley Crl : CD (SD) rats of both genders (five weeks old) were obtained from OrientBio Co., Ltd. (Seoungnam, Korea). The animals were allowed to acclimatize for one week before the initiation of experiments. Animals were housed in a stainless wire cage (255 mm W × 465 mm L × 200 mm H) at ≤5 animals/cage for the quarantine period and at ≤2 animals/cage for the observation period. Pelleted food was purchased from PMI Nutrition International (USA). The pellet food was gamma-ray irradiated and given *ad libitum*. Tap water was given *ad libitum*, following UV irradiation and filtration. The water was analyzed at the Daejeon Regional Institute of Health and Environment (Daejeon, Korea). The animal room was maintained at a temperature of 22 ± 3°C, a relative humidity of 50 ± 10%, an air ventilation frequency of 10~20 times/hr, a light intensity of 150~300 Lux, and a 12-hr light/dark cycle (08:00~20:00). The study was conducted according to the guidance of the Institutional Animal Care and Use Committee at the Korea Institute of Toxicology, KRICT (accredited by AAALAC International 1998). The numbers of approval for the acute and subchronic toxicity studies were G08109 and G08111, respectively.

### 2.4. Group Assignment and Treatment

YMJ is widely used as a traditional medicine and was not anticipated to cause animal death following a single dose. The highest dose of 2000 mg/kg, with a common ratio of 2, was selected for this study. YMJ extract was dissolved in distilled water for injection (Choongwae Pharmaceutical, Ltd., Korea). Distilled water was given to the animals in the vehicle control group. The animals were grouped into four groups with five rats/sex/group at random, and YMJ was dissolved in purified water and administered by oral gavage at doses of 0, 500, 1000, or 2000 mg/kg body weight. The observations of general condition, toxic symptoms, and mortality in rats were monitored for 14 days after YMJ administration. The grouping of animals in good health during the acclimation period was carried out using the A-module of Path/Tox System (Ver. 4.2.2, Xybion Medical Systems Corporation, USA).

In the subchronic study, rats (10 rats/sex/group) were randomly divided into four groups. A previous four-week repeated dose study showed no abnormal changes even at the highest dose, 2000 mg/kg. Therefore, 2000 mg/kg was selected as the highest dose, with a common ratio of 2.

### 2.5. Observation and Examination

#### 2.5.1. Acute Oral Toxicity in Rats

Mortality and clinical signs were continually observed for 0, 1, 2, 3, 4, 5, and 6 hr after dosing on day 1 and at one time per day from day 2 to day 15. The animals were weighed before dosing (day 1) and after dosing (days 2, 4, 8, and 15) using an electronic balance (Sartorious Co., Germany). On day 15, all animals were euthanized by exsanguination from the abdominal aorta and abdominal vena cava under CO_2_ gas overdose and examined for internal organ abnormalities.

#### 2.5.2. Subchronic 13-Week Oral Toxicity in Rats

Mortality and clinical signs were recorded once a day during the study period using the Path/Tox Program (Ver. 4.2.2). Animals were weighed on animal arrival, randomization, the first day of dosing, once a week thereafter, and at the termination of the experiment. The amounts of food and water given and their remnants on the next day were measured to calculate the difference, which was regarded as daily consumption. External eye examination was investigated during the pretest period; both external and fundus examinations were completed using an indirect binocular ophthalmoscope (Vantage Plus Digital, Keeler Ltd., UK). 

During the last week of dosing, urinalysis was carried out with urine collected over approximately 16 hr from all animals. Urinalysis measurements included urine volume, specific gravity, pH, protein, ketone body, occult blood, glucose, bilirubin, nitrite, urobilinogen, color, and sediment (cast, epithelial cell, erythrocyte, and leucocyte). Urine volume was measured using a mass cylinder, color with macroscopic examination, and sediment using a microscope. The other parameters in the urinalysis were analyzed using Multistix 10 SG strips (Bayer, USA) and a CliniTek-100 (Bayer, USA).

Animals were fasted over night prior to necropsy and blood sampling. All living animals were anesthetized by isoflurane inhalation, blood samples were taken, and then the animals were sacrificed by exsanguination from the aorta. Complete gross observation was performed on all terminated animals. The blood samples were collected into tubes containing EDTA-2K and analyzed for complete blood cell counts. Complete blood cells were measured by an ADVIA120 Hematology System (Bayer, USA). Prothrombin time (PT) and activated partial thromboplastin time (APTT) were analyzed with blood samples treated with 3.2% sodium citrate using an ACL 9000 (Instrumentation Laboratory, Italy).

Blood samples for serum biochemistry and hematology were drawn from the posterior vena cava of all animals. Serum samples were prepared after blood centrifugation and analyzed using a Toshiba 200FR NEO (Toshiba Co., Japan). The following parameters were measured: aspartate aminotransferase (AST), alanine aminotransferase (ALT), alkaline phosphatase (ALP), blood urea nitrogen (BUN), creatinine (CREA), glucose (GLU), total cholesterol (TCHO), albumin/globulin ratio (A/G), total protein (TP), albumin (ALB), creatine kinase (CK), triglyceride (TG), total bilirubin (TBIL), phospholipid (PL), gamma glutamyl transferase (GGT), calcium (Ca), inorganic phosphorus (IP), chloride (Cl), sodium (Na), and potassium (K) [12].

Absolute organ weights were measured, and relative organ weights (organ/body weight) were calculated for the organs of all animals when sacrificed.

### 2.6. Statistical Analysis

Data collected during the study were examined for variance homogeneity using the Bartlett's test. When the Bartlett's test indicated no significant deviations from variance homogeneity, a one-way ANOVA was performed at *α* = 0.05. When significance was noted, a multiple comparison test (Dunnett's test) was conducted to determine which groups were significantly different. In the case that significant deviations from variance homogeneity were observed, a nonparametric comparison test (Kruskal-Wallis test) was conducted. When a significant difference was observed in the Kruskal-Wallis test, the Dunn's Rank Sum test was conducted to perform pairwise comparisons between pairs. Statistical analyses were performed using the Path/Tox System (Ver. 4.2.2). The level of significance used was *P* < .05 or 0.01. Because treatment-related animal deaths were not observed, the LD_50_ was not measured.

## 3. Results

### 3.1. Acute Oral Toxicity in Rats

**Figure 2:**
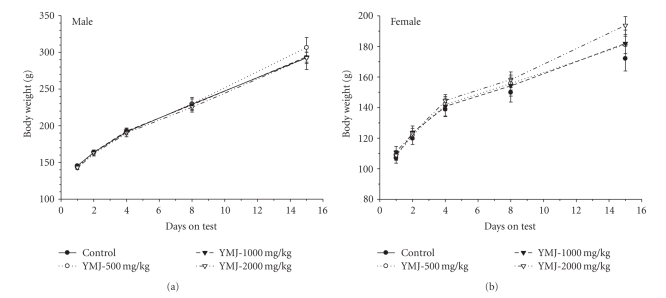
Changes in body weight during a two-week acute toxicity study. (a) Male rats and (b) female rats.

There were no animal deaths in any groups. Therefore, the approximate lethal doses of YMJ extract in male and female rats are higher than 2000 mg/kg. Clinical signs observed included loss of fur in 1/5 males from day 11 until the end of the analysis and 2/5 females from day 4 until the end of the analysis in the YMJ 2000 mg/kg group. Normal body weight gains were observed in males and females of all dose groups (Figure 2). No significant differences were observed between the vehicle control and YMJ treatment groups. No abnormal gross findings were observed in any animals.

### 3.2. Subchronic 13-Week Oral Toxicity in Rats

There were no animal deaths during the study period. In males, clinical signs observed during the study period included loss of fur (1/10) and swollen pinna (1/10) in the vehicle control group; loss of fur (1/10), swollen pinna (1/10), and scratch wound (1/10) in the 500 mg/kg group; loss of fur (4/10) and scratch wound (2/10) in the 1000 mg/kg group; loss of fur (2/10) and scratch wound (2/10) in the 2000 mg/kg group. In the females, clinical signs included loss of fur (1/10) in the vehicle control group; loss of fur (4/10) and scratch wound (1/10) in the 500 mg/kg group; loss of fur (2/10) in the 1000 mg/kg group; loss of fur (3/10) in the 2000 mg/kg group.

**Figure 3:**
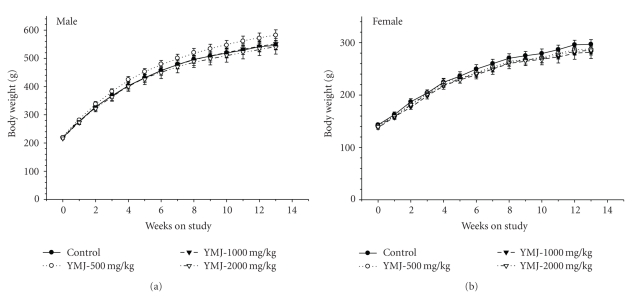
Increase in body weight during the 13-week subchronic toxicity study. (a) Male rats and (b) female rats.

**Figure 4:**
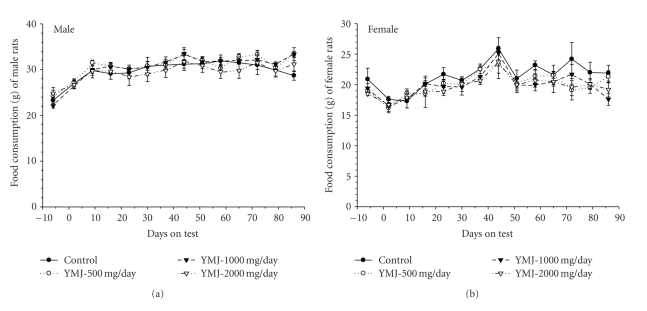
Food consumption of rats administrated *Yukmijihwang-tang *orally for 13 weeks. (a) Male rats and (b) female rats.

No treatment-related body weight changes were observed during the study period (Figure 3). An increase (16%) in food consumption measured at day 86 was observed in the 500 and 1000 mg/kg groups compared to the vehicle control group (Figure 4). The overall water consumption of animals receiving the YMJ extract was generally similar to that of the vehicle control (data not shown).

In ophthalmological examination, no abnormalities were noted in any animals. No treatment-related changes were observed in urinalysis during the study period. Hematological examination performed at necropsy showed an increase in NEU% (50%) and a decrease in LYM% (9%) in the male 1000 mg/kg group (Table 2). Serum biochemical examination performed at female necropsy showed a decrease in AST (20%) in the 1000 mg/kg group and a decrease in TBIL (14%) in the 2000 mg/kg group (Table 3).

Gross findings at male necropsy included swollen pinna (1/10) and irregular surface of lung (1/10) in the vehicle control group, swollen pinna (1/10) in the 500 mg/kg group, small testes and epididymis (1/10) in the 1000 mg/kg group, and a scratched wound on face (1/10) in the 2000 mg/kg group. In females, gross findings included focus/foci in the adrenal glands (1/10) in the vehicle control group and nodule in the kidneys (1/10), adhesion in the diaphragm (1/10), hemorrhage in the thoracic cavity (1/10), and discoloration of the thymus (1/10) in the 2000 mg/kg group.

Organ weights measured at female necropsy showed a decrease (33%) in the absolute weight of the uterus/cervix in the 500 mg/kg group and an increase (9%) in the relative weight of the liver in the 2000 mg/kg group (Table 4). 

In histopathological examination, lesions such as inflammatory cell foci in the liver and kidneys, mineralization in the kidneys, and cardiomyopathy in the heart in males and females in the 2000 mg/kg groups were also detected in the vehicle control group with similar incidence rates. The acanthosis and chondropathy in the ear, chronic dermatitis of the skin, M-nephroblastoma in the kidney, pigmented macrophages in the thymus, tubular atrophy in the testes, and aspermia in the epididymides were not dose dependent and occurred at low incidence rates. There were other lesions that were considered to be spontaneous or nontreatment related.

## 4. Discussion

Globally, the increasing use of complementary and alternative medicine (CAM) such as herbal remedies may be due to a variety of factors, including the limits of current therapy and adverse effects of conventional drugs [13]. Traditional herbal medicine is the most important part of CAM and has been utilized for treating diseases and promoting the health of humans for thousands of years; in addition, it has become a popular alternative choice conventional medicine. Although there are many traditional herbal medicine interventions available, and some have been verified by clinical trials, their efficacy and safety are still questioned by consumers [14]. 

YMJ is one of the most popular herbal formulas in Asian countries; however, evidence-based information is limited. Many studies have reported pharmacological efficacies and benefits of YMJ, but there is no information on its risk and safety such as its acute and subchronic toxicity. 

To evaluate the acute toxicity, YMJ extract was orally given at doses 0, 500, 1000, or 2000 mg/kg to male and female Crl:CD (SD) rats. Mortalities, clinical signs, body weight changes, and gross findings were monitored for 14 days (days 1~15). The loss of fur observed in some animals at the highest dose group (2000 mg/kg) has been observed in control SD rats also and considered to be due to individual variation. 

No treatment-related toxicity was observed in the study carried out to evaluate the subchronic 13-week repeated oral dose toxicity of YMJ extract. Some changes noted in the clinical observation, food consumption, hematology, serum biochemistry, gross findings, and organ weights were considered to be not treatment-related but incidental and within normal ranges. Histopathological findings, such as the inflammatory cell foci in the liver and kidneys, mineralization in the kidneys, and cardiomyopathy in the heart were also observed in the vehicle control group at similar levels and incidence rates, indicating no toxicological significance. Lesions such as acanthosis and chondropathy in the ear, chronic dermatitis of the skin, tubular atrophy in the testes, and aspermia in the epididymides were considered to be associated with gross findings but appear to be incidental because of a lack of dose dependency and low incidence rates. There were other lesions that were considered to be spontaneous or not related to the treatments.

Consequently, no acute toxicity was found in rats treated with YMJ extract, and approximate lethal doses for males and females were higher than 2000 mg/kg. The 13-week repeated oral administration of YMJ in SD rats showed no toxicity and a no-observed-adverse-effect-level (NOAEL) of 2000 mg/kg/day.

In humans, the reported single dose of YMJ is about 46.875 g dried herb [1], which is equivalent to 12.656 g of the extract (yield of extract = 27.0% of the raw material). Considering the average body weight of an adult as 60 kg, the dose for a 60 kg human is 211 mg of YMJ extract/kg. To convert the human dose to rat dose equivalent, the amount would be 1,329 mg/kg (211 mg/kg × 6.3) according to van Miert [15]. The dose equivalent per kg for rat (6.3) was derived by dividing the Km factor, body surface area (m^2^) to body weight (kg) ratio, for humans with the Km factor for rat [15]. The amount of YMJ extract, 1,329 mg, is a value less than the NOAEL (2000 mg/kg in rats).

In conclusion, the present studies demonstrate that at doses consumed in traditional medicine, the aqueous extract of YMJ may be relatively safe because it did not cause any lethality or changes in the general behavior in rats in both the acute and subchronic toxicity studies.

## Figures and Tables

**Table 1:** Composition of Yukmijihwang-tang (YMJ).

Herbs	Scientific names	Ratio
Rehmanniae Radix Preparat	*Rehmannia glutinosa* Liboschitz var. purpurea Makino	8
Dioscoreae Rhizoma	*Dioscorea batatas* Decaisne	4
Corni Fructus	*Cornus officinalis* Siebold et Zuccarini	4
Hoelen	*Poria cocos* Wolf	3
Moutan Cortex	*Paeonia suffruticosa* Andrews	3
Alismatis Rhizoma	*Alisma orientale* Juzepczuk	3

**Table 2:** Hematological parameters of male and female rats on subchronic toxicity study.

	(a)* Male *				(b)* Female *			
Parameter	Vehicle control	YMJ-500 mg	YMJ-1000 mg	YMJ-2000 mg	Vehicle control	YMJ-500 mg	YMJ-1000 mg	YMJ-2000 mg
WBC (×10^3^/*μ*L)	12.10 ± 1.04	10.72 ± 1.06	10.49 ± 0.48	10.83 ± 0.58	8.74 ± 0.71	8.54 ± 0.72	7.56 ± 0.71	9.33 ± 0.91
Reticulocytes (%)	2.1 ± 0.1	2.3 ± 0.2	2.0 ± 0.1	2.3 ± 0.1	1.9 ± 0.1	1.7 ± 0.1	1.9 ± 0.1	2.3 ± 0.1
Neutrophils (%)	14.46 ± 1.39	18.57 ± 1.82	21.65 ± 1.76**	18.37 ± 1.10	13.07 ± 2.25	13.27 ± 1.57	15.91 ± 0.74	14.98 ± 2.31
Lymphocytes (%)	77.8 ± 1.6	74.3 ± 1.9	71.0 ± 1.7**	75.1 ± 1.2	80.4 ± 2.2	79.2 ± 1.7	76.9 ± 0.8	79.1 ± 2.2
Eosinophils (%)	1.1 ± 0.1	1.1 ± 0.2	1.2 ± 0.1	1.1 ± 0.2	1.2 ± 0.2	1.2 ± 0.2	1.2 ± 0.1	1.1 ± 0.1
Monocytes (%)	4.7 ± 0.4	4.5 ± 0.3	4.5 ± 0.3	3.6 ± 0.3	3.6 ± 0.3	4.6 ± 0.7	4.0 ± 0.3	3.3 ± 0.2
Basophils (%)	0.7 ± 0.1	0.7 ± 0.1	0.6 ± 0.1	0.7 ± 0.1	0.7 ± 0.1	0.7 ± 0.1	0.9 ± 0.1	0.7 ± 0.1
LUC (%)	1.3 ± 0.1	0.9 ± 0.1	1.0 ± 0.1	1.1 ± 0.2	1.0 ± 0.1	0.9 ± 0.1	1.1 ± 0.2	0.9 ± 0.1
RBC (×10^6^/*μ*L)	8.63 ± 0.16	8.65 ± 0.13	8.70 ± 0.14	8.66 ± 0.11	8.19 ± 0.09	8.20 ± 0.13	8.38 ± 0.11	7.92 ± 0.09
Hemoglobin (g/dL)	15.7 ± 0.4	16.1 ± 0.1	16.1 ± 0.3	16.4 ± 0.1	16.1 ± 0.3	16.0 ± 0.3	16.3 ± 0.2	15.9 ± 0.2
Hmatocrit (%)	44.6 ± 1.0	45.4 ± 0.5	45.4 ± 0.7	46.0 ± 0.5	44.9 ± 0.6	44.1 ± 0.7	45.3 ± 0.4	43.8 ± 0.5
MCV (fL)	51.7 ± 0.8	52.5 ± 0.9	52.2 ± 0.7	53.1 ± 0.5	54.9 ± 0.5	53.8 ± 0.5	54.1 ± 0.4	55.4 ± 0.6
MCH (pg)	18.2 ± 0.3	18.7 ± 0.3	18.5 ± 0.3	19.0 ± 0.2	19.7 ± 0.2	19.5 ± 0.2	19.5 ± 0.2	20.1 ± 0.2
MCHC (g/dL)	35.2 ± 0.1	35.5 ± 0.2	35.4 ± 0.2	35.8 ± 0.2	35.9 ± 0.2	36.3 ± 0.2	36.0 ± 0.3	36.3 ± 0.3
Platelet (×10^3^/*μ*L)	1048 ± 24	1034 ± 36	1053 ± 36	1065 ± 49	1089 ± 48	1080 ± 48	1098 ± 20	1051 ± 38
PT (sec)	16.1 ± 0.4	15.5 ± 0.3	16.0 ± 0.4	16.8 ± 0.6	14.5 ± 0.2	14.3 ± 0.2	14.2 ± 0.3	14.1 ± 0.1
APTT (sec)	18.6 ± 0.4	18.8 ± 0.6	18.2 ± 0.4	18.8 ± 0.3	15.0 ± 0.2	14.8 ± 0.5	15.3 ± 0.4	15.1 ± 0.4

Values are mean ± SEM for 10 rats in each group. **, significant differences from the vehicle control (0) group (*P* < .01). WBC: white blood cells; LUC: large unstained cells; RBC: red blood cells; MCV: mean corpuscular volume; MCH: mean corpuscular hemoglobin; MCHC: mean corpuscular hemoglobin concentration; PT: prothrombin time; APTT: activated partial prothrombin time.

**Table 3:** Serum biochemical parameters of male and female rats on subchronic toxicity study.

	(a)* Males *				(b)* Females *			
Parameter	Vehicle control	YMJ-500 mg	YMJ-1000 mg	YMJ-2000 mg	Vehicle control	YMJ-500 mg	YMJ-1000 mg	YMJ-2000 mg
Glucose (mg/dL)	120.1 ± 6.9	118.8 ± 4.6	117.2 ± 5.5	110.6 ± 4.4	113.8 ± 10.4	107.2 ± 6.5	117.4 ± 5.3	107.6 ± 10.0
BUN (mg/dL)	13.3 ± 0.8	13.8 ± 0.5	13.8 ± 0.5	13.9 ± 0.7	16.8 ± 0.7	15.7 ± 0.8	16.3 ± 0.8	16.3 ± 0.7
Creatinine (mg/dL)	0.51 ± 0.02	0.54 ± 0.01	0.51 ± 0.01	0.53 ± 0.01	0.63 ± 0.02	0.59 ± 0.00	0.60 ± 0.02	0.61 ± 0.03
Total protein (g/dL)	6.85 ± 0.14	6.75 ± 0.09	6.79 ± 0.13	6.80 ± 0.12	7.16 ± 0.17	7.23 ± 0.11	7.10 ± 0.19	7.25 ± 0.16
Albumin (g/dL)	4.25 ± 0.06	4.23 ± 0.04	4.26 ± 0.05	4.27 ± 0.05	4.67 ± 0.10	4.71 ± 0.07	4.59 ± 0.11	4.70 ± 0.09
A/G (ratio)	1.64 ± 0.04	1.69 ± 0.04	1.69 ± 0.05	1.69 ± 0.03	1.90 ± 0.05	1.87 ± 0.04	1.83 ± 0.03	1.85 ± 0.04
TCHO (mg/dL)	66.2 ± 8.2	67.8 ± 4.2	66.6 ± 3.7	64.9 ± 5.8	72.0 ± 4.2	73.7 ± 4.0	71.2 ± 4.6	73.2 ± 5.6
Triglyceride (mg/dL)	53.1 ± 8.0	57.3 ± 9.7	58.6 ± 11.7	49.1 ± 6.9	43.3 ± 5.1	42.5 ± 4.2	37.3 ± 4.3	36.1 ± 3.3
Phospholipid (mg/dL)	104 ± 9	104 ± 6	104 ± 5	100 ± 8	139 ± 7	138 ± 7	137 ± 8	134 ± 10
AST (IU/dL)	107.8 ± 4.1	103.6 ± 6.0	101.6 ± 7.1	103.1 ± 6.1	133.3 ± 7.2	116.3 ± 5.9	106.6 ± 4.1**	113.7 ± 7.8
ALT (IU/dL)	33.8 ± 1.5	30.4 ± 0.9	32.5 ± 2.5	31.0 ± 0.9	36.3 ± 2.7	31.5 ± 1.3	30.6 ± 3.2	28.6 ± 1.8
TBIL (mg/dL)	0.140 ± 0.005	0.149 ± 0.007	0.132 ± 0.004	0.134 ± 0.003	0.158 ± 0.006	0.147 ± 0.007	0.145 ± 0.005	0.136 ± 0.004*
ALP (IU/dL)	196.1 ± 6.8	199.1 ± 15.3	216.9 ± 13.9	222.6 ± 18.0	103.6 ± 4.5	105.4 ± 10.1	124.9 ± 14.6	86.0 ± 5.7
CK (IU/dL)	472 ± 46	449 ± 50	459 ± 75	401 ± 45	553 ± 56	517 ± 40	418 ± 34	552 ± 59
Ca (mg/dL)	10.90 ± 0.15	10.85 ± 0.08	10.80 ± 0.17	10.86 ± 0.13	11.31 ± 0.22	11.13 ± 0.19	11.05 ± 0.25	11.21 ± 0.18
IP (mg/dL)	10.05 ± 0.26	9.27 ± 0.27	9.23 ± 0.31	9.55 ± 0.31	8.02 ± 0.49	8.16 ± 0.37	7.99 ± 0.38	8.25 ± 0.48
Na (mg/dL)	144 ± 0	144 ± 1	146 ± 1	145 ± 1	144 ± 0	145 ± 0	145 ± 1	144 ± 1
K (mg/dL)	8.84 ± 0.42	8.12 ± 0.52	8.00 ± 0.56	9.15 ± 0.61	7.72 ± 0.19	7.03 ± 0.20	7.32 ± 0.13	7.16 ± 0.31
Cl (mg/dL)	101 ± 1	101 ± 0	102 ± 1	102 ± 0	103 ± 1	103 ± 1	103 ± 0	102 ± 1
GGT (mg/dL)	0.00 ± 0.00	0.00 ± 0.00	0.00 ± 0.00	0.00 ± 0.00	0.00 ± 0.00	0.25 ± 0.26	0.01 ± 0.01	0.02 ± 0.02

Values are mean ± SEM for 10 rats in each group. *: significant differences from the vehicle control (0) group (*P* < .05). **: significant differences from the vehicle control (0) group (*P* < .01). BUN: blood urea nitrogen; A/G: albumin/globulin; TCHO: total cholesterol, AST: aspartate aminotransferase; ALT: alanine aminotransferase; TBIL: total bilirubin; CK: creatine kinase; ALP: alkaline phosphatase; IP: inorganic phosphorus; GGT: gammaglutamyl transferase.

**Table 4:** Absolute organ weights (g) of male and female rats on subchronic toxicity study.

	(a) *Males *				(b)* Females *			
Parameter	Vehicle control	YMJ-500 mg	YMJ-1000 mg	YMJ-2000 mg	Vehicle control	YMJ-500 mg	YMJ-1000 mg	YMJ-2000 mg
Brain	2.08 ± 0.03	2.15 ± 0.03	2.10 ± 0.04	2.07 ± 0.03	1.95 ± 0.03	1.90 ± 0.02	1.88 ± 0.02	1.91 ± 0.02
Pituitary	0.013 ± 0.001	0.012 ± 0.001	0.012 ± 0.001	0.013 ± 0.001	0.017 ± 0.001	0.014 ± 0.001	0.015 ± 0.001	0.016 ± 0.001
Liver	13.85 ± 0.73	14.45 ± 0.58	14.08 ± 0.72	13.85 ± 0.79	7.35 ± 0.23	6.95 ± 0.30	6.99 ± 0.32	7.62 ± 0.21
Spleen	0.810 ± 0.069	0.842 ± 0.045	0.763 ± 0.038	0.765 ± 0.043	0.520 ± 0.018	0.466 ± 0.031	0.480 ± 0.027	0.500 ± 0.027
Adrenal gland	0.064 ± 0.002	0.062 ± 0.004	0.059 ± 0.002	0.063 ± 0.003	0.072 ± 0.004	0.065 ± 0.002	0.065 ± 0.003	0.068 ± 0.002
Kidneys	3.51 ± 0.14	3.41 ± 0.13	3.42 ± 0.11	3.42 ± 0.15	1.90 ± 0.05	1.80 ± 0.06	1.89 ± 0.09	1.89 ± 0.26
Heart	1.58 ± 0.05	1.56 ± 0.06	1.55 ± 0.04	1.55 ± 0.07	0.98 ± 0.02	0.93 ± 0.04	0.89 ± 0.03	0.95 ± 0.03
Thymus	0.381 ± 0.027	0.450 ± 0.027	0.418 ± 0.040	0.404 ± 0.023	0.348 ± 0.026	0.319 ± 0.032	0.314 ± 0.027	0.324 ± 0.024
Salivary glands	0.710 ± 0.020	0.773 ± 0.023	0.701 ± 0.021	0.732 ± 0.037	0.426 ± 0.011	0.429 ± 0.018	0.436 ± 0.023	0.425 ± 0.015
Lung	1.57 ± 0.04	1.67 ± 0.05	1.60 ± 0.05	1.59 ± 0.09	1.20 ± 0.02	1.19 ± 0.04	1.14 ± 0.03	1.23 ± 0.04
Thyroid/Parathyriod	0.025 ± 0.002	0.024 ± 0.002	0.025 ± 0.002	0.023 ± 0.002	0.015 ± 0.002	0.014 ± 0.001	0.015 ± 0.001	0.014 ± 0.001
Epididymides	1.52 ± 0.03	1.45 ± 0.04	1.48 ± 0.06	1.50 ± 0.04	—	—	—	—
Seminal vesicle	1.80 ± 0.10	1.83 ± 0.07	1.75 ± 0.07	1.97 ± 0.09	—	—	—	—
Prostate	0.726 ± 0.039	0.692 ± 0.036	0.705 ± 0048	0.745 ± 0.038	—	—	—	—
Testes	3.49 ± 0.11	3.23 ± 0.07	3.26 ± 0.18	3.34 ± 0.06	—	—	—	—
Ovaries	—	—	—	—	0.088 ± 0.004	0.078 ± 0.004	0.079 ± 0.004	0.084 ± 0.028
Uterus/Cervix	—	—	—	—	0.830 ± 0.078	0.556 ± 0.049*	0.847 ± 0.083	0.638 ± 0.097

Values are mean ± SEM for 10 rats in each group. *: significant differences from the vehicle control (0) group (*P* < .05). **: significant differences from the vehicle control (0) group (*P* < .01).
